# Youth E-Cigarette Use and Functionally Important Respiratory Symptoms: The Population Assessment of Tobacco and Health (PATH) Study Waves 3 and 4

**DOI:** 10.3390/ijerph192215324

**Published:** 2022-11-19

**Authors:** Elizabeth R. Stevens, Shu Xu, Raymond Niaura, Charles M. Cleland, Scott E. Sherman, Andi Mai, Emma Karey, Nan Jiang

**Affiliations:** 1 Department of Population Heath, Grossman School of Medicine, New York University, New York, NY 10016, USA; 2 School of Global Public Health, New York University, New York, NY 10012, USA; 3 Department of Medicine, VA New York Harbor Healthcare System, New York, NY 10010, USA; 4 Department of Medicine, Grossman School of Medicine, New York University, New York, NY 10016, USA

**Keywords:** tobacco, e-cigarettes, youth, respiratory health

## Abstract

Respiratory effects of e-cigarette use among youth are not fully understood. This study investigated the longitudinal association between e-cigarette use and a validated index of functionally important respiratory symptoms among US youth. Data from Waves 3–4 of the Population Assessment of Tobacco and Health Study were analyzed. The sample included youth (aged 12–17) without asthma at baseline (Wave 3), who completed a follow-up survey (Wave 4), and were not missing data for analytic variables (n = 3899). Exposure was e-cigarette use status (never, former, or current) at baseline. The outcome was a respiratory symptom index based on responses for seven wheezing items at Wave 4. An index of ≥2 was defined as having functionally important respiratory symptoms. Lagged logistic regression models examined the association between baseline e-cigarette use and functionally important respiratory symptoms at follow-up by combustible tobacco use status (never or ever), and controlling for baseline covariates. At baseline, 13.7% of participants reported former e-cigarette use, and 4.3% reported current use. Baseline e-cigarette use did not increase the odds of having functionally important respiratory symptoms at follow-up regardless of combustible tobacco use status. Future research on larger populations of e-cigarette users with longer follow-up periods will improve our understanding of the respiratory risks associated with e-cigarette use among youth.

## 1. Introduction

While the rate of pediatric e-cigarette use in the United States (US) is down significantly from its peak (from 27.5% in 2019 to 14.1% in 2022 in high school; from 10.5% in 2019 to 3.3% in 2022 in middle school) [1,2], 2.55 million American middle- and high-school students still use e-cigarettes [1]. E-cigarettes remain the most commonly used tobacco product among youth in the US, with an increasing proportion of tobacco initiation attributable to e-cigarettes [3]. Growing evidence links e-cigarette use with negative respiratory health outcomes in youth [4,5], which is of particular concern since their lungs have not yet fully matured. E-cigarette use can interfere with lung development and impair respiratory immunity, as well as increase oxidative stress and inflammation [6,7]. Frequent use of e-cigarettes among youth has been associated with increased odds of respiratory symptoms (e.g., coughing and wheezing) [4], and respiratory complications (e.g., asthma exacerbations, pneumonia, epiglottitis, bronchitis, and acute respiratory distress) [5].

Previous studies assessing the respiratory consequences of e-cigarette use among youth have significant limitations: Most used cross-sectional data. Only one study to date has assessed the longitudinal effect of e-cigarette use on wheezing in the past 12 months with a nationally representative sample of US youth using the Population Assessment of Tobacco and Health (PATH) data (Waves 3 and 4) [8]. That study concluded that, among youth with no self-reported asthma, e-cigarette use at baseline (Wave 3) was not associated with increased adjusted odds of wheezing at 1-year follow-up (Wave 4). However, extrapolation of these findings is limited by several experimental conditions: (1) it did not control for baseline respiratory symptoms (which could affect reported respiratory outcomes at follow-up), (2) it failed to capture other respiratory symptoms indicative of pulmonary impairment (i.e., dry cough), which could lead to an underestimation of any respiratory impacts of e-cigarette use, and (3) it did not test the combined risk of e-cigarette use and combustible tobacco use on wheezing, despite combustible tobacco use being an established risk factor for respiratory diseases [9].

An index of functionally important respiratory symptoms (hereafter referred to as the “respiratory symptom index”) has been generated through the validation of the seven-question International Study of Asthma and Allergies in Children (ISAAC) wheezing module from items in a PATH adult cohort [10,11,12]. The index has been recommended as a respiratory outcome for studies (both cross-sectional and longitudinal) assessing the effects of tobacco use on respiratory health. The respiratory symptom index has not been previously used to assess respiratory symptoms among youth who use e-cigarettes. In this study, using the respiratory symptom index, we sought to investigate the longitudinal association between e-cigarette use at baseline and respiratory changes at one-year follow-up in a PATH youth cohort by combustible tobacco use status. 

## 2. Methods

### 2.1. Study Population

Data were analyzed from the Waves 3–4 Public Use File of the PATH Study, a national longitudinal cohort study of tobacco use and related health status in a representative household sample of US adults and youth aged ≥12 years. The PATH Study was launched in 2011 with a purpose of informing and monitoring the impact of the Food and Drug Administration’s regulatory actions on reducing mortalities and morbidities attributed to tobacco use [13]. Wave 3 and 4 surveys were collected in October 2015–October 2016 (Wave 3) and December 2016–January 2018 (Wave 4) using a 4-stage stratified area probability sample design [14]. More details about the PATH Study are available elsewhere [13]. This study sample included 3,899 youth (aged 12–17 years) who completed both waves of surveys, had no missing data on any analytic variables, and had not been diagnosed with asthma at Wave 3 (responded “no” to the question “Have you ever been told by a doctor, nurse or other health professional that you have asthma?”).

### 2.2. Key Measures

#### 2.2.1. Respiratory Symptom Index (Waves 3 and 4)

The PATH Study includes a core, validated wheezing module from the International Study of Asthma and Allergies in Children [10,11]. The module includes 7 questions which have been widely used in adolescent populations globally. An index of functionally important respiratory symptoms (hereafter referred to as the “respiratory symptom index”) has been generated through the validation of the 7 items in a PATH adult cohort (with no respiratory disease such as chronic obstructive pulmonary disease (COPD)) [12].

Following prior research that created and validated the respiratory symptom index [12], we calculated the index based on 7 questions (“Have you ever had wheezing or whistling in the chest at any time in the past? [Yes/No]”; “Have you had wheezing or whistling in the chest in the past 12 months? [Yes/No]”; “How many attacks of wheezing have you had in the past 12 months? [‘None’, ‘1–3′, ‘4–12′, ‘More than 12′]”; “In the past 12 months, how often, on average, has your sleep been disturbed due to wheezing? [‘Never’, ‘Less than one night per week’, ‘One or more nights per week’]”; “In the past 12 months, has wheezing ever been severe enough to limit your speech to only one or two words between breaths? [Yes/No]”; “In the past 12 months, has your chest sounded wheezy during or after exercise? [Yes/No]”; “In the past 12 months, have you had a dry cough at night? [Yes/No]”). The respiratory symptom index ranges from 0 (answering “No” to all dichotomous items and selecting lowest levels for multiple-answer items) to 9 (answering “Yes” to all dichotomous items and selecting highest levels for multiple-answer items). 

An index score of <2 was defined as having no functionally important respiratory symptoms, and an index of ≥2 as having functionally important respiratory symptoms. The cut-off value was recommended in the prior study of a PATH adult cohort that concluded the threshold values of ≥2 and ≥3 were associated with functional pulmonary impairment [12]. We chose the more sensitive cut point (≥2) in an effort to capture more subtle functional changes present in youth who are likely to have less cumulative exposure than adult users [15].

#### 2.2.2. E-Cigarette Use and Combustible Tobacco Use (Wave 3)

Participants were asked a series of questions about e-cigarette use and were classified as never, former, or current e-cigarette users. Individuals with no history of electronic nicotine product use were deemed “never users.” Individuals who reported a history of electronic nicotine product use, but not in the past 30 days, were classified as “former users.” Current e-cigarette users reported past 30-day use of an electronic nicotine product.

Similarly, participants were asked a series of questions regarding the use of combustible tobacco products including cigarettes, traditional cigars, cigarillos, filtered cigars, pipes, and hookah. Due to the small sample size of current (past 30-day) combustible tobacco users, current users (n = 151; 4.0%) and former users (n = 430; 11.1%) were combined as ever users (n = 581; 15.0%). Thus, participants were dichotomized as never or ever combustible tobacco users. Never users reported they had never used any combustible tobacco products, and ever users reported having used a combustible tobacco product. 

#### 2.2.3. Covariates (Wave 3)

Covariates included age group (12–15 years old and 16–17 years old), gender (male and female), race/ethnicity (non-Hispanic White, non-Hispanic Black, Hispanic, and other), parental education attainment (less than high school, GED, high school graduate, some college or associate degree, Bachelor’s degree, and advanced degree), household income in past 12-month (<$50,000 and ≥$50,000), body mass index (BMI; underweight, normal weight, overweight, and obese), past 7-day secondhand smoke exposure (“During the past 7 days, about how many hours were you around others who were smoking? Include time in your home, in a car, at school, or outdoors” with a numeric response from the participant), and chronic disease (“Have you ever been told by a doctor, nurse or other health professional that you had high blood pressure? [Yes/No]”; “Have you ever been told by a doctor, nurse or other health professional that you had high cholesterol? [Yes/No]”; “Have you ever been told by a doctor, nurse or other health professional that you had diabetes, sugar diabetes, high blood sugar, or borderline diabetes? [Yes/No]”). Participants were considered as having no chronic disease if they answered “No” to all 3 questions, and otherwise as having a chronic disease.

### 2.3. Statistical Analysis 

We performed data analyses using SAS version 9.4 (SAS Institute Inc., Cary, NC, USA). PATH sample weights were used in all analyses to account for survey sampling frame, nonresponse, and selection bias. We conducted descriptive statistics to summarize baseline (Wave 3) demographics, clinical characteristics, and tobacco use status of the entire sample and by their combustible tobacco use status (never and ever use). Rao–Scott chi-square tests (for categorical variables) and ANOVA (for continuous variables) compared the difference between never and ever combustible tobacco users. Next, separate lagged logistic regression models were used to examine the association between e-cigarette use at baseline (Wave 3) and the respiratory symptom index (≥2 vs. <2) at follow-up (Wave 4) by combustible tobacco use status, controlling for baseline covariates (i.e., sociodemographics, secondhand smoke exposure, BMI, chronic disease, and respiratory symptom index). 

## 3. Results 

Our sample was evenly split by gender (Table 1). Most (65.3%) were aged 16–17 years old and 56.0% were non-Hispanic White. One-third (30.9%) of participants’ parents completed some college or an associate degree, and 56.3% had a household income of less than $50,000. Participants reported an average of 2.36 h (SE = 0.18) being exposed to secondhand smoke in the past 7 days. Most were normal weight (60.0%) with no health professional diagnosis of chronic disease (93.6%). At Wave 3, 18.0% of participants reported they had ever used an e-cigarette, including 4.3% current users, and 15.0% had used a combustible tobacco product. E-cigarette use was more prevalent among combustible tobacco users (*p* < 0.001).

At Wave 3, participants reported an average respiratory symptom index of 0.62 (SE = 0.02). Ever combustible tobacco users reported higher index scores than never combustible tobacco users (0.76, SE = 0.05 vs. 0.60, SE = 0.02; *p* = 0.003). Overall, 14.2% of participants reported a respiratory symptom index of ≥2 (data not reported in tables). Ever combustible tobacco users were more likely to report an index of ≥2 than never combustible tobacco users (16.9% vs. 13.8%; *p* = 0.029). At Wave 4, participants reported an average respiratory symptom index of 0.59 (SE = 0.02), and 13.1% reported an index of ≥2 (data not reported in tables). Ever combustible tobacco users were more likely to report a respiratory symptom index of ≥2 than never combustible tobacco users (17.0% vs. 12.4%, *p* = 0.001).

E-cigarette use (including former and current) was not associated with higher odds of a respiratory symptom index of ≥2, adjusting for baseline respiratory symptom index and other covariates. The same was true despite history of combustible tobacco use (among never combustible tobacco users: ever e-cigarette use vs. never use: adjusted odds ratio (AOR) = 1.20, 95% confidence interval (CI): 0.78–1.85, *p* = 0.441; current e-cigarette use vs. never use: AOR = 0.86, 95% CI: 0.32–2.32, *p* = 0.767; among ever combustible tobacco users: ever e-cigarette use vs. never use: AOR = 1.13, 95% CI: 0.57–2.23, *p* = 0.725; current e-cigarette use vs. never use: AOR = 0.55, 95% CI: 0.18–1.61, *p* = 0.270; Table 2). 

Higher baseline respiratory symptom index scores were associated with higher adjusted odds of reporting a respiratory symptom index of ≥2. This was observed among all youth regardless of combustible tobacco use status (among never combustible tobacco users: AOR = 2.19, 95% CI: 1.97–2.44, *p* < 0.001; among ever combustible tobacco users: AOR = 2.06, 95% CI: 1.59–2.66, *p* < 0.001). Of ever combustible tobacco users, Hispanics and non-Hispanic others were less likely to have a respiratory symptom index of ≥2 at follow-up compared to non-Hispanic Whites (Hispanic: AOR = 0.41, 95% CI: 0.19–0.89, *p* = 0.024; non-Hispanic other: AOR = 0.10, 95% CI: 0.01–0.88, *p* = 0.038). Parental education of high school graduate was associated with an increased adjusted odd of having a respiratory symptom index of ≥2 compared to those with some college or associate degrees (AOR = 1.66, 95% CI: 1.01–2.74, *p* = 0.047).

## 4. Discussion

This is the first study investigating the longitudinal effect of e-cigarette use with a validated, composite measure of functionally important respiratory symptoms using a nationally representative sample of US youth. Among asthma-free youth, e-cigarette use at baseline did not increase odds of the development of functionally important respiratory symptoms (respiratory symptom index ≥2) at one-year follow-up. This was also observed among never and ever combustible tobacco users. These results are consistent with a previous longitudinal study that did not find an association between e-cigarette use and wheezing among youth in the PATH cohort between Waves 3 and 4 [8]. Interestingly, outcomes from both longitudinal studies are inconsistent with those from cross-sectional studies. A cross-sectional study of youth from PATH Wave 3 found a statistically significant association between e-cigarette use and an increased odd of dry cough and wheezing symptoms [4]. Several other cross-sectional studies of youth in southern California [16], Hong Kong [17], and South Korea [18] have also reported significant associations between e-cigarette use and respiratory symptoms (e.g., coughing and wheezing) and disease (e.g., asthma). The significant association found in cross-sectional data, but not in our longitudinal analyses, may be attributable to the potential presence of sample selection bias. By limiting the analysis cohort to those without asthma at baseline, our study sample was relatively healthy and any individuals who had already developed symptoms were eliminated, therefore potentially underestimating the effect of e-cigarette use. In addition, our models controlled for baseline respiratory symptom index which was shown to be a strong predictor of reporting a subsequent index of ≥2 and therefore, the finding of no associations detected reflected no new important respiratory symptom developed within one-year period between baseline and follow-up.

The lack of detected association between e-cigarette use and the development of functionally important respiratory symptoms in our analysis may be related to the small number of youth using e-cigarettes (4.3% were current users: n = 168) and the brief period between baseline and follow up (approximately one year). While acute e-cigarette exposures can induce cellular toxicity [19], and transient changes in cardiovascular parameters [20], sustained changes in respiratory function may take longer to develop and present, particularly among youth with no respiratory health issues at baseline and who may have had a limited total lifetime exposure to e-cigarettes. Research is warranted with a longer follow-up period and larger sample of e-cigarette users to further examine the respiratory risk associated with e-cigarette use among youth.

This study has several limitations. First, self-reported data are subject to recall bias and reporting errors. Second, respondents with asthma at Wave 3 were excluded. However, youth with other respiratory diseases (e.g., bronchitis) were not excluded, due to data availability. The public-use PATH data for youth only contains the measure of self-reported diagnosis of asthma and did not include measures of other respiratory diseases. This may have impacted the sensitivity of testing the respiratory effects of e-cigarette use. Third, although this study investigated the longitudinal association between baseline e-cigarette use and respiratory outcome at follow-up, our findings should not be interpreted as evidence for the causal relationship between e-cigarette use and respiratory health. Fourth, our findings were based on data collected between 2015 and 2018. The COVID-19 pandemic and recent regulations on e-cigarette devices (e.g., U.S. Food and Drug Administration’s marketing authorizations and flavor restrictions) may have impacted e-cigarette usage, highlighting the need for further investigation on the effects of e-cigarette use on respiratory health outcomes. Finally, the outcome itself–respiratory symptom index –and the cut-off value have only been validated in PATH adult respondents [12]. However, a more sensitive cut-off point (≥1) was tested in our analysis, yielding similar results. Whether this is due to a latency between tobacco use and detectable respiratory changes, being underpowered due to the small sample size of youth e-cigarette users, or the insensitivity of this index for use in pediatric populations, is unknown.

Strengths of this study are the longitudinal design using data collected from a large nationally representative sample of youth. Additionally, use of an index made up of a compact seven-item core wheezing assessment for children as the primary respiratory outcome should capture even early evidence of pulmonary dysfunction.

## 5. Conclusions

E-cigarette use was not significantly associated with odds of developing functionally important respiratory symptoms at one-year follow up after adjusting for baseline respiratory symptom index and other factors among asthma-free youth. Future studies with longer follow-up periods and larger cohorts of e-cigarette users are needed to better characterize any respiratory risks that e-cigarettes may pose to youth.

## Figures and Tables

**Table 1:** Characteristics of youth sample of the PATH Study Wave 3.

	Total Sample (n = 3899)		Never Combustible Tobacco Users (n = 3318; 85.0%)		Ever Combustible Tobacco Users (n = 581; 15.0%)		*p*
	n	(%)	n	(%)	n	(%)	
Gender							0.111
Female	1956	(50.1)	1671	(50.4)	285	(48.6)	
Male	1943	(49.9)	1647	(49.6)	296	(51.4)	
Age group (in years)							<0.001
12–15	1337	(34.7)	1218	(37.2)	119	(20.5)	
16–17	2562	(65.3)	2100	(62.8)	462	(79.5)	
Race/ethnicity							0.579
Non-Hispanic White	1894	(56.0)	1604	(55.9)	290	(56.6)	
Non-Hispanic Black	511	(13.1)	438	(12.9)	73	(13.3)	
Hispanic	1152	(21.8)	982	(21.7)	170	(22.6)	
Others	342	(9.1)	294	(9.5)	48	(7.5)	
Parental education level							<0.001
Less than high school	567	(11.7)	463	(10.9)	104	(16.2)	
GED	157	(3.6)	125	(3.3)	32	(5.4)	
High school graduate	724	(17.1)	598	(16.6)	126	(19.7)	
Some college or associate degree	1212	(30.9)	1000	(29.9)	212	(37.1)	
Bachelor’s degree	770	(22.3)	708	(24.0)	62	(12.7)	
Advanced degree	469	(14.4)	424	(15.3)	45	(8.9)	
Household income							<0.001
<$50,000	1986	(56.3)	1753	(58.3)	233	(44.6)	
≥$50,000	1829	(41.7)	1494	(39.7)	335	(53.2)	
Unreported	84	(2.0)	71	(2.0)	13	(2.2)	
Body mass index							<0.001
Under weight	581	(15.8)	531	(17.2)	50	(8.0)	
Normal	2303	(60.0)	1961	(60.0)	342	(60.1)	
Overweight	638	(15.3)	527	(14.8)	111	(18.3)	
Obese	377	(8.9)	299	(8.0)	78	(13.6)	
Secondhand smoke exposure, M (±SE)	2.36	(±0.18)	1.81	(±0.18)	5.53	(±0.64)	<0.001
Chronic disease							0.074
No	3632	(93.6)	3104	(93.9)	528	(91.5)	
Yes	267	(6.4)	214	(6.1)	53	(8.5)	
Respiratory symptom index, M (±SE)	0.62	(±0.02)	0.60	(±0.02)	0.76	(±0.05)	0.003
E-cigarette use status							<0.001
Never use	3182	(82.0)	2998	(90.8)	184	(31.9)	
Former use	549	(13.7)	266	(7.5)	283	(48.6)	
Current use	168	(4.3)	54	(1.7)	114	(19.5)	

Note. GED: general equivalency diploma.

**Table 2:** Association between baseline e-cigarette use and functionally important respiratory symptom index of ≥2 at follow-up by combustible tobacco use status among youth.

	Among Never Combustible Tobacco Users			Among Ever Combustible Tobacco Users		
	AOR	[95% CI]	*p*	AOR	[95% CI]	*p*
E-cigarette use status						
Never use (ref)	1.00			1.00		
Former use	1.20	[0.78, 1.85]	0.411	1.13	[0.57, 2.23]	0.725
Current use	0.86	[0.32, 2.32]	0.767	0.55	[0.18, 1.61]	0.270
Gender						
Female (ref)	1.00			1.00		
Male	0.95	[0.75, 1.19]	0.637	1.17	[0.62, 2.20]	0.624
Age group (in years)						
12–15 (ref)	1.00			1.00		
16–17	0.95	[0.73, 1.22]	0.670	0.95	[0.47,1.89]	0.874
Race/ethnicity						
Non-Hispanic White (ref)	1.00			1.00		
Non-Hispanic Black	0.71	[0.45, 1.13]	0.143	1.36	[0.59, 3.11]	0.464
Hispanic	0.77	[0.54, 1.11]	0.154	0.41	[0.19, 0.89]	0.024
Other	0.65	[0.40, 1.08]	0.097	0.10	[0.01, 0.88]	0.038
Parental education level						
Less than high school	0.86	[0.50, 1.48]	0.579	0.68	[0.16, 2.89]	0.594
GED	1.04	[0.53, 2.02]	0.918	2.13	[0.46, 10.00]	0.333
High school graduate	1.08	[0.75, 1.54]	0.691	1.66	[1.01, 2.74]	0.047
Some college or associate degree (ref)	1.00			1.00		
Bachelor’s degree	0.77	[0.56, 1.07]	0.115	1.71	[0.65, 4.53]	0.274
Advanced degree	0.85	[0.54, 1.34]	0.479	1.76	[0.66, 4.72]	0.260
Household income						
<$50,000	0.82	[0.61, 1.10]	0.185	1.27	[0.68, 2.37]	0.442
≥$50,000 (ref)	1.00			1.00		
Unreported	0.44	[0.14, 1.40]	0.160	0.83	[0.07, 9.81]	0.884
Secondhand smoke exposure	1.00	[0.99, 1.01]	0.482	1.01	[0.99, 1.03]	0.298
Body mass index						
Under weight	0.70	[0.45, 1.07]	0.099	1.35	[0.53, 3.44]	0.529
Normal (ref)	1.00			1.00		
Overweight	1.20	[0.89, 1.63]	0.230	1.25	[0.61, 2.53]	0.538
Obese	1.39	[0.88, 2.22]	0.158	1.37	[0.60, 3.12]	0.446
Chronic disease						
No (ref)	1.00			1.00		
Yes	1.58	[0.99, 2.53]	0.057	1.09	[0.34, 3.50]	0.879
Baseline respiratory symptom index	2.19	[1.97, 2.44]	<0.001	2.06	[1.59, 2.66]	<0.001

Notes. AOR: adjusted odds ratio; CI: confidence interval; GED: general equivalency diploma.

## Data Availability

The data used in this study is public and available online: Public Use File data of the Population Assessment of Tobacco and Health Study (PATH).

## References

[1] Cooper M., Park-Lee E., Ren C., Cornelius M., Jamal A., Cullen K.A. (2022). Notes from the Field: E-cigarette Use Among Middle and High School Students-United States, 2022. MMWR. Morb. Mortal. Wkly. Rep..

[2] Wang T.W., Neff L.J., Park-Lee E., Ren C., Cullen K.A., King B.A. (2020). E-cigarette Use Among Middle and High School Students-United States, 2020. MMWR. Morb. Mortal. Wkly. Rep..

[3] Stanton C.A., Sharma E., Seaman E.L., Kasza K.A., Edwards K.C., Halenar M.J., Taylor K.A., Day H., Anic G., Hull L.C. (2020). Initiation of any tobacco and five tobacco products across 3 years among youth, young adults and adults in the USA: Findings from the PATH Study Waves 1–3 (2013–2016). Tob. Control.

[4] Cherian C., Buta E., Simon P., Gueorguieva R., Krishnan-Sarin S. (2021). Association of Vaping and Respiratory Health among Youth in the Population Assessment of Tobacco and Health (PATH) Study Wave 3. Int. J. Environ. Res. Public Health.

[5] Tzortzi A., Kapetanstrataki M., Evangelopoulou V., Behrakis P. (2020). A Systematic Literature Review of E-Cigarette-Related Illness and Injury: Not Just for the Respirologist. Int. J. Environ. Res. Public Health.

[6] Miyashita L., Foley G. (2020). E-cigarettes and respiratory health: The latest evidence. J. Physiol..

[7] Lyzwinski L.N., Naslund J.A., Miller C.J., Eisenberg M.J. (2022). Global youth vaping and respiratory health: Epidemiology, interventions, and policies. NPJ Prim. Care Respir. Med..

[8] Tackett A.P., Keller-Hamilton B., Smith C.E., Hébert E.T., Metcalf J.P., Queimado L., Stevens E.M., Wallace S.W., McQuaid E.L., Wagener T.L. (2020). Evaluation of Respiratory Symptoms Among Youth e-Cigarette Users. JAMA Netw. Open.

[9] US Department of Health & Human Services (2014). The Health Consequences of Smoking—50 Years of Progress: A Report of the Surgeon General.

[10] Pearce N., Aït-Khaled N., Beasley R., Mallol J., Keil U., Mitchell E., Robertson C. (2007). Worldwide trends in the prevalence of asthma symptoms: Phase III of the International Study of Asthma and Allergies in Childhood (ISAAC). Thorax.

[11] Asher M.I., Keil U., Anderson H.R., Beasley R., Crane J., Martinez F., Mitchell E.A., Pearce N., Sibbald B., Stewart A.W. (1995). International Study of Asthma and Allergies in Childhood (ISAAC): Rationale and methods. Eur. Respir. J..

[12] Halenar M.J., Sargent J.D., Edwards K.C., Woloshin S., Schwartz L., Emond J., Tanski S., Pierce J.P., Taylor K.A., Lauten K. (2021). Validation of an Index for Functionally Important Respiratory Symptoms among Adults in the Nationally Representative Population Assessment of Tobacco and Health Study, 2014–2016. Int. J. Environ. Res. Public Health.

[13] Hyland A., Ambrose B.K., Conway K.P., Borek N., Lambert E., Carusi C., Taylor K., Crosse S., Fong G.T., Cummings K.M. (2017). Design and methods of the Population Assessment of Tobacco and Health (PATH) Study. Tob. Control.

[14] U.S. Department of Health and Human Services; National Institutes of Health; National Institute on Drug Abuse; U.S. Department of Health and Human Services Food and Drug Administration Center for Tobacco Products Population Assessment of Tobacco and Health (PATH) Study [United States] Public-Use Files (ICPSR 36498). https://www.icpsr.umich.edu/web/NAHDAP/studies/36498.

[15] Landrigan P.J., Kimmel C.A., Correa A., Eskenazi B. (2004). Children’s health and the environment: Public health issues and challenges for risk assessment. Environ. Health Perspect..

[16] McConnell R., Barrington-Trimis J.L., Wang K., Urman R., Hong H., Unger J., Samet J., Leventhal A., Berhane K. (2017). Electronic Cigarette Use and Respiratory Symptoms in Adolescents. Am. J. Respir. Crit. Care Med..

[17] Wang M.P., Ho S.Y., Leung L.T., Lam T.H. (2016). Electronic Cigarette Use and Respiratory Symptoms in Chinese Adolescents in Hong Kong. JAMA Pediatr..

[18] Cho J.H., Paik S.Y. (2016). Association between Electronic Cigarette Use and Asthma among High School Students in South Korea. PLoS ONE.

[19] Knapp S. (2020). Vaping: Cell Damage at the Receiving ENDS. Am. J. Respir. Cell Mol. Biol..

[20] Wehrli F.W., Caporale A., Langham M.C., Chatterjee S. (2020). New Insights from MRI and Cell Biology Into the Acute Vascular-Metabolic Implications of Electronic Cigarette Vaping. Front. Physiol..

